# Abnormal Cerebral Blood Flow and Volumetric Brain Morphometry in Patients With Obstructive Sleep Apnea

**DOI:** 10.3389/fnins.2022.934166

**Published:** 2022-07-06

**Authors:** Ping Xiao, Kelei Hua, Feng Chen, Yi Yin, Jurong Wang, Xiangjun Fu, Jiasheng Yang, Qingfeng Liu, Queenie Chan, Guihua Jiang

**Affiliations:** ^1^The Second School of Clinical Medicine, Southern Medical University, Guangzhou, China; ^2^Department of Otolaryngology-Head & Neck Surgery, Guangdong Second Provincial General Hospital, Guangzhou, China; ^3^Department of Medical Imaging, Guangdong Second Provincial General Hospital, Guangzhou, China; ^4^Department of Respiratory and Critical Care Medicine, Center for Sleep Medicine, Guangdong Second Provincial General Hospital, Guangzhou, China; ^5^Philips Healthcare, Hong Kong, Hong Kong SAR, China

**Keywords:** obstructive sleep apnea, pseudo-continuous arterial spin labeling, cerebral blood flow, voxel-based morphometry, Montreal Cognitive Assessment

## Abstract

Obstructive sleep apnea (OSA) is a serious breathing disorder, leading to myocardial infarction, high blood pressure, and stroke. Brain morphological changes have been widely reported in patients with OSA. The pathophysiological mechanisms of cerebral blood flow (CBF) changes associated with OSA are not clear. In this study, 20 patients with OSA and 36 healthy controls (HCs) were recruited, and then pseudo-continuous arterial spin labeling (pCASL) and voxel-based morphometry (VBM) methods were utilized to explore blood perfusion and morphological changes in the patients with OSA. Compared with the HC group, the OSA group showed increased CBF values in the right medial prefrontal cortex (mPFC), left precentral gyrus, and right insula and showed decreased CBF values in the right temporal pole (TP) and the right cerebellum_Crus2. Compared with the HC group, the patients with OSA showed decreased gray matter volume (GMV) in the right dorsal lateral prefrontal cortex (DLPFC), the right occipital pole, and the vermis. There were no significantly increased GMV brain regions found in patients with OSA. Pearson correlation analysis showed that the reduced GMV in the right DLPFC and the right occipital pole was both positively correlated with Mini-Mental State Examination (MMSE) (*r* = 0.755, *p* < 0.001; *r* = 0.686, *p* = 0.002) and Montreal Cognitive Assessment (MoCA) scores (*r* = 0.716, *p* = 0.001; *r* = 0.601, *p* = 0.008), and the reduced GMV in the right occipital pole was negatively correlated with duration of illness (*r* = −0.497, *p* = 0.036). Patients with OSA have abnormal blood perfusion metabolism and morphological changes in brain regions including the frontal lobe and the cerebellum and were closely related to abnormal behavior, psychology, and cognitive function, which play an important role in the pathophysiological mechanism of OSA.

## Introduction

Obstructive sleep apnea (OSA) is a frequently but insufficiently recognized breathing disorder, associated with prominent comorbidities, especially cerebrovascular and cardiovascular diseases such as high blood pressure, myocardial infarction, and stroke (Arzt et al., 2005; Yaggi et al., 2005; Chang et al., 2014; Lamberts et al., 2014). Obstructive sleep apnea can cause sleep fragmentation, daytime sleepiness, reduced work performance, increased risk of traffic accidents, and reduced quality of life (Bennett et al., 1999). The deficits in attention, memory, and visuoconstructive abilities frequently accompany OSA. Intermittent episodes of hypoxia during sleep and significant hemodynamic changes during apnea are considered to be central to the pathophysiology of the development of ischemic brain injury (Pizza et al., 2012; Winklewski and Frydrychowski, 2013). Previous studies have reported morphological brain changes that could underlie those symptoms in patients with OSA (Macey et al., 2002; Morrell et al., 2003; Canessa et al., 2011; O’Donoghue et al., 2012). However, the pathophysiological mechanisms of cerebral blood flow (CBF) changes associated with OSA are not fully understood.

During the past three decades, neuroimaging studies explored abnormal functional metabolism neural regions of various medical disorders such as OSA, by analyzing CBF and metabolic processes. A considerable number of studies focus on the CBF and the continuous positive air pressure therapy for the possible improvement of CBF in OSA. CBF is controlled by a variety of automatic regulatory mechanisms, including chemical, metabolic, and neurogenic regulation, with changes in carbon dioxide and oxygen (to a lesser extent) being the most powerful stimuli leading to changes in cerebrovascular flow. Acute hypoxia stimulates cerebral vasodilation and increases CBF as a compensatory mechanism. Single-photon emission computed tomography (SPECT) and positron emission tomography (PET) have been previously used for the observed brain hemometabolic process. However, SPECT and PET scans take a long time, need to inject invasive radiotracers, cause strong ionizing radiation, and have a low spatial resolution of SPECT and PET images. Compared with SPECT and PET, pseudo-continuous arterial spin labeling (pCASL) is a recent non-invasive MRI technique that can rapidly quantify local CBF using endogenous contrast agents instead of invasive radiotracers and has a high temporal and spatial resolution. Resting CBF is closely related to brain metabolisms such as glucose utilization, oxygen consumption, and aerobic glycolysis. Resting CBF indicator was widely used in bipolar disorder, major depressive disorder, schizophrenia, and multiple sclerosis to identify abnormal hemometabolic brain regions. However, no studies have focused on abnormal CBF changes in OSA using the pCASL technique.

In addition, voxel-based morphometry (VBM) is a widely used neuroimaging technique for measuring changes in gray matter and white matter volumes (GMV and WMV) in a non-invasive and region-specific manner. It is also an automatic whole-brain method that can calculate the local volume of gray matter in an unbiased manner. Several previous VBM studies found abnormal GMV brain regions in OSA. However, these results are inconsistent such as gray matter loss in the frontal and parietal cortex (Canessa et al., 2011), anterior cingulate (Macey et al., 2012), hippocampus (Morrell et al., 2003; Canessa et al., 2011), and cerebellum (Macey et al., 2002). Moreover, Fergal et al., found no GMV deficits nor focal structural changes in severe OSA (O’Donoghue et al., 2005). These differences may be related to the differences in methodologies, study population, or disease severity. However, with continuous positive airway pressure (CPAP) therapy, most of the VBM differences in GMV in patients with OSA were observed to be reversible, possibly due to changes in neuronal blood flow caused by changes in neurons or angiogenesis (Canessa et al., 2011; O’Donoghue et al., 2012). Furthermore, previous studies adopted a separate CBF method or VBM method to investigate OSA, abnormal CBF is closely related to GMV changes (Gonchigsuren et al., 2022), and therefore more efforts should be made to concentrate on CBF and VBM imaging modalities to explore the common changes and specific changes of CBF and VBM and to identify whether abnormal changes in GMV are the basis of abnormal CBF or abnormal CBF leading to abnormal GMV in OSA.

Thus, we aimed to obtain an understanding of the correlation between hemodynamic differences and morphological abnormalities in patients with OSA by using pCASL and VBM imaging and to detect shared and specific patterns of neuronal blood flow and GMV in OSA. The characteristic altered patterns of CBF and GMV may play important roles in the pathophysiological mechanism of OSA.

## Materials and Methods

### Participants

A total of 20 outpatients diagnosed with moderate-to-severe OSA [apnea-hypopnea index (AHI) > 15 events/h] were recruited from the Center for Sleep Medicine of Second People’s Hospital of Guangdong Province. And 36 healthy control sex-, age-, and education-matched participants as healthy controls (HCs) (AHI < 5) were recruited through local advertisements. All participants were right-handed and drug-naive. The exclusion criteria included the presence of severe lung, heart, or kidney disease; history of mental retardation, neurological disorders, organic brain disorder, or any comorbid somatic disorders; body mass index (BMI) > 40 kg/m^2^; malignant disease; pregnancy or breastfeeding; use of antidepressants, hypnotics, morphine, other respiratory-depressant medication, or non-psychiatric drugs; comorbidity with any other Axis-I mental disorders or Axis-II personality disorder; any clinical sign of previous stroke or transient ischemic attack; and drink more than 14 units per week. During the three days before the imaging period, the Epworth Sleepiness Scale (ESS), which is a validated questionnaire, was used to evaluate subjective daytime sleepiness in the context of sleep disorders for each participant. The Mini-Mental State Examination (MMSE) was published more than 30 years ago in 1975 as a practical method of grading cognitive impairment (Folstein et al., 1975). The Montreal Cognitive Assessment (MoCA) is an increasingly popular cognitive screening tool that has good sensitivity and specificity in detecting cognitive impairment and includes an assessment of multiple cognitive domains (Shi et al., 2018). The MMSE and MoCA were used to assess the psychological state and cognitive impairment of each participant.

This study was approved by the Ethics Committee of the Second People’s Hospital of Guangdong Province, and the ethics approval was given in March 2022. This study was carried out in accordance with the World Medical Association’s Declaration of Helsinki. All participants agreed in writing to participate in this study.

### Image Acquisition

Imaging data were performed using a 3.0 T MRI scanner (Ingenia; Philips, Best, The Netherlands). The pCASL sequence was used for three-dimensional (3D) fast spin-echo acquisition and background suppression in the resting-state perfusion imaging. The acquisition parameters were the following: repetition time (TR) = 4,155 ms, echo time (TE) = 33 ms, post-labeling delay (PLD) = 2,000 ms, field of view (FOV) = 240 × 240 mm^2^, in-plane matrix = 64 × 59, in-plane voxel size = 3.75 × 3.75 × 6.00, slice thickness/gap = 6.0/0 mm, 20 axial slices covering the whole brain, number of signals averaged (NSA) = 1, and acquisition time = 4 min 51 s. In addition, a 3D T1-weighted brain volume imaging sequence covering the whole brain was used for structural data acquisition with the following: TR/TE = 7.8/3.6 ms, flip angle = 8°, slice thickness/gap = 1.0/0 mm, FOV = 240 × 240 mm, matrix = 256 × 256, NSA = 1, and acquisition time = 5 min 56 s. Routine MRI images were also collected to exclude anatomical abnormalities. All participants were found by two experienced radiologists to confirm that there were no brain structural abnormalities.

### Cerebral Blood Flow Processing

The pCASL images were analyzed on a Philips post-processing workstation. Quantification of CBF was calculated using the following equation:


$$\mathrm{CBF}=\frac{6000\,\lambda \,\left({\mathrm{SI}}_{\mathrm{control}}-{\mathrm{SI}}_{\mathrm{label}}\right)\,e^{\frac{\mathrm{PLD}}{T_{1,\mathrm{blood}}}}}{2\,\alpha \,T_{1,\mathrm{blood}}\,{\mathrm{SI}}_{\mathrm{PD}}\,\left(1-e^{-\frac{\tau }{T_{1,\mathrm{blood}}}}\right)}\,\left[\mathrm{ml}/100\,g/\min\right],$$

where T1 of blood (T_1,blood_) was assumed to be 1,650 ms at 3.0T, partition coefficient (λ) 0.9, labeling efficiency (α) 0.85, labeling duration (τ) 1,800 ms, and PLD 2,000 ms. SI_*control*_ and SI_*label*_ are the time-averaged signal intensities in the control and label images, respectively, SI_*PD*_ is the signal intensity of a proton density-weighted image. Using the Statistical Parametric Mapping (SPM12^1^) software standardizes the CBF maps into a standard Montreal Neurological Institute (MNI) space: (1) the individual 3D T1-weighted structure images and the individual CBF brain maps were co-registered to obtain the individual T1’ brain maps; (2) all the individual T1’ brain maps were non-linearly normalized to T1 template in standard space; (3) using the normalization parameters estimated in step (2), all the CBF images were normalized to MNI space and resampled to the voxel size of 2 × 2 × 2 mm^3^. (4) For standardization, the z-transform was performed on the CBF value of each voxel: zCBF = (single voxel CBF - mean CBF of whole-brain)/standard deviation of the whole brain CBF. (5) The zCBF maps were smoothed using a Gaussian smoothing kernel with a half maximum and full width (FWHM) of 6 mm.

### Voxel-Based Morphometry Processing

All T1-weighted brain structure images were processed using CAT12^2^ based on the SPM12 software^3^. For the VBM analysis, diffeomorphic anatomical registration through an exponentiated Lie algebra algorithm (DARTEL) (Ashburner, 2007) was used to improve the quality of structural image registration (Klein et al., 2009). The preprocessing process of structural MRI data is as follows: (1) the original individual T1-weighted images were segmented into white matter (WM), gray matter (GM), and cerebrospinal fluid (CSF). (2) After segmenting GM and WM images of all subjects, a study-specific template was created using DARTEL. (3) The individual segmented images were warped to a special template for the study and spatially normalized to the MNI space using modulation. (4) The modulated GM images were smoothed using a Gaussian smoothing kernel with an FWHM of 6 mm.

### Statistical Analysis

SPSS for the Windows software, version 19.0 (SPSS Inc., Chicago, IL, United States) was performed for statistical analyses. The Kolmogorov-Smirnov test was used to evaluate the normality of age, years of education, total intracranial volume (TIV), BMI, AHI, MMSE, MoCA, and ESS. In addition, age, years of education, TIV, BMI, AHI, MMSE, MoCA, and ESS follow the normal distribution. The independent-sample *t*-test was used to compare age, years of education, TIV, BMI, AHI, MMSE, MoCA, and ESS between the OSA and HC groups. 𝒳^2^ test was used to compare the gender differences between two groups. All tests were two-tailed, and *p* < 0.05 was considered statistically significant.

For comparisons of CBF and VBM maps, a voxel-based comparison of CBF maps and VBM maps was performed between OSA and HCs groups using two-sample *t*-tests, with individuals’ age, sex, years of education, and TIV as nuisance covariates. A family wise error (FWE) cluster-level correction for multiple comparisons (*p* < 0.001, cluster level *p* < 0.05, cluster > 165 voxels) was used in all group comparisons.

Once the clusters showing significant group differences in CBF and VBM maps between two groups were identified, the cluster was saved as a binary mask to extract the CBF and GMV values. Then, we further calculated the Pearson’s correlation coefficients between these CBF and GMV values and the MMSE, MoCA, ESS, and duration of illness. The Bonferroni correction was used for multiple comparisons.

## Results

### Demographic Information

Table 1 shows the demographic information and clinical characteristics of all recruited participants. Two patients with OSA were excluded from further analyses due to image artifacts. Two experienced radiologists confirmed that all participants have no brain structural abnormalities from routine MRI images. Finally, 18 patients with OSA and 36 HCs were included. There were no significant differences in age, education, and sex between two groups. The TIV, BMI, AHI, and ESS of patients with OSA were greater than those in HCs. The scores of MMSE and MoCA in the OSA group were lower than those in the HC group.

**TABLE 1:** Demographic and clinical data and (standard deviations) by group.

	OSAS	HCs	*p* values
Number of subjects	18	36	
Age (years)	43.00 (15.73)	40.61 (12.01)	0.538*
Education (years)	13.50 (3.47)	13.42 (3.36)	0.933*
Sex (male/female)	13/5	27/9	0.826^†^
TIV (mm^3^)	1514.61 (93.54)	1439.58 (126.68)	0.031*
BMI (kg/m^2^)	25.42 (3.86)	21.49 (1.50)	< 0.001*
AHI (times/min)	37.86 (25.96)	2.26 (1.01)	< 0.001*
MMSE	28.28 (1.56)	29.36 (0.83)	< 0.001*
MoCA	27.56 (2.06)	29.08 (1.20)	< 0.001*
ESS	10.33 (4.59)	3.14 (1.59)	< 0.001*
Duration of illness (years)	13.72 (13.55)	n/a	

Means (with standard deviations in parentheses) are reported unless otherwise noted. OSAS, Obstructive sleep apnea syndrome; HCs, healthy controls; TIV, total intracranial volume; BMI, Body Mass Index; AHI, apnea-hypopnea index; MMSE, Mini-mental State Examination; MoCA, Montreal Cognitive Assessment; ESS, Epworth Sleepiness Scale. *The p values were obtained by independent-sample t-tests. ^†^The p value for sex distribution was obtained by chi-square test.

### Differences in Cerebral Blood Flow Values Between Two Groups

**FIGURE 1:**
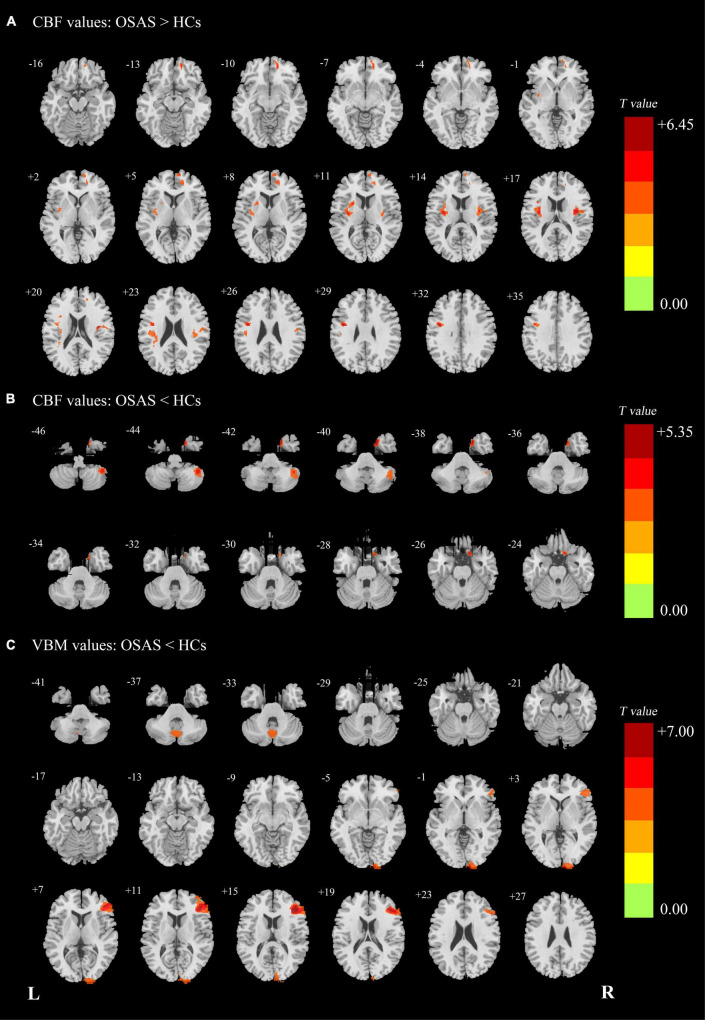
**(A)** Patients with OSA showed significantly increased CBF clusters than HCs. **(B)** Patients with OSA showed significantly decreased cerebral CBF clusters than HCs. **(C)** Patients with OSA showed significantly decreased VBM clusters than HCs. OSA, obstructive sleep apnea syndrome; HCs, healthy controls; CBF, cerebral blood flow; VBM, voxel-based morphometry; L (R), left (right) hemisphere.

Compared with the HC group, the OSA group showed increased CBF values in the right medial prefrontal cortex (mPFC), left precentral gyrus (extending to the left insula and left putamen), and right insula (extending to the right rolandic_oper) and showed decreased CBF values in the right TP and the right cerebellum_Crus2 (Figures 1A,B and Table 2).

**TABLE 2:** The areas of significantly different CBF and VBM values between the OSAS patients and the HCs. (*p* < 0.001, cluster level *p* < 0.05, cluster > 165, FWE corrected).

Brain regions	Extending regions	Brodman Area	Montreal Neurological Institute Coordinates			Peak *t* Value	Cluster Size (voxel numbers)
			X	Y	Z		
**CBF values: OSAS > HCs**							
R mPFC		11	14	52	−10	5.23	262
L precentral gyrus		6	−42	−2	30	6.43	606
	L insula	48	−33	−9	16	5.11	
	L putamen	NA	−23	8	10	4.285	
R insula		48	36	−6	18	5.66	244
	R rolandic_oper	48	37	−4	16	5.07	
**CBF values: OSAS < HCs**							
R TP		36	20	7	−40	5.31	165
R cerebellum_Crus2		NA	45	−49	−44	4.195	202
**VBM values: OSAS < HCs**							
R DLPFC		45	42	35	17	6.65	2494
R occipital pole		17	14	−101	2	4.21	1163
Vermis		NA	−3	−68	−35	4.46	541

*CBF, cerebral blood flow; VBM, voxel Based morphometry; OSAS, obstructive sleep apnea syndrome; HCs, healthy controls; FWE, family wise error; mPFC, medial prefrontal cortex; TP, temporal pole; DLPFC, dorsal lateral prefrontal cortex; R, right hemisphere.*

### Differences in Voxel-Based Morphometry Values Between Two Groups

Compared with the HC group, the patients with OSA showed decreased GMV in the right DLPFC, the right occipital pole, and the vermis. There were no significantly increased GMV brain regions found in patients with OSA (Figure 1C and Table 2).

### Correlation Analysis

**FIGURE 2:**
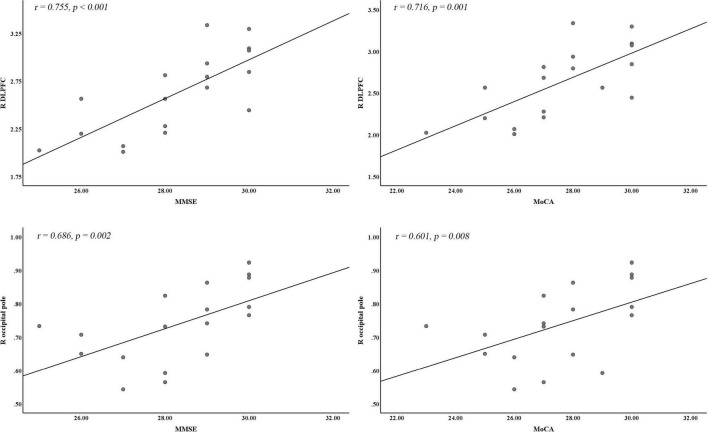
Correlation analysis between significantly different CBF or VBM clusters and cognitive and clinical variables. DLPFC, dorsal lateral prefrontal cortex; MMSE, Mini-Mental State Examination; MoCA, Montreal Cognitive Assessment; R, right hemisphere.

Pearson correlation analysis showed that the reduced GMV in the right DLPFC and the right occipital pole was positively correlated with both MMSE (*r* = 0.755, *p* < 0.001; *r* = 0.686, *p* = 0.002) and MoCA scores (*r* = 0.716, *p* = 0.001; *r* = 0.601, *p* = 0.008), and the reduced GMV in the right occipital pole was negatively correlated with duration of illness (*r* = −0.497, *p* = 0.036) (*p* < 0.05/32 = 0.0016, Bonferroni corrected) (Figure 2). There were no significant correlations between the left precentral gyrus, right insula, right TP, right cerebellum_Crus2, and vermis and the MMSE, MoCA, ESS, and duration of illness.

## Discussion

To the best of our knowledge, this is the first study to combine VBM and CBF modalities to detect the pathophysiological mechanism in patients with OSA. The results showed that, for VBM modality, compared with HCs, the patients with OSA showed decreased GMV in the right DLPFC, the right occipital pole, and the vermis. The reduced GMV in the right DLPFC and the right occipital pole was positively correlated with both MMSE and MoCA scores, and the reduced GMV in the occipital pole was negatively correlated with the duration of illness. For CBF modality, compared with HCs, the patients with OSA showed decreased CBF values in the right TP and the right cerebellum_Crus2 and increased CBF values in the right mPFC, the left precentral gyrus, and the right insula. All the results suggested that disturbed brain volume and blood flow mainly located in the frontal lobe, cerebellum, primary functional area (including precentral gyrus and occipital pole), insula, and TP and were closely related to the psychological problems and cognitive deficits.

In this study, in OSA, GMV reductions and CBF increases were observed in the right DLPFC and the right mPFC, respectively. In addition, the reduced GMV in OSA was positively correlated with MMSE and MoCA scores. The prefrontal cortex, particularly DLPFC, is the area of the brain that controls various executive functions, such as behavioral inhibition, environmental switching, emotional self-regulation, and arousal (Beebe and Gozal, 2002). The mPFC is the hub of the default mode network (DMN) and is generally more active at rest than during cognitive activity. Recently, the executive dysfunction associated with OSA cannot be explained by sleepiness itself (Harrison and Horne, 2000) but may be a manifestation of neuronal damage in the prefrontal cortex (including DLPFC and mPFC) (Nofzinger, 2005). A previous VBM meta-analysis reported GMV reductions in the frontal lobe in OSA (Shi et al., 2017), and Bai et al. (2021) found decreased fractional amplitude of low-frequency fluctuation and regional homogeneity in the prefrontal cortex in childhood OSA, which supported our findings. In DMN (such as mPFC), changes in functional connectivity and regional brain activity were observed in resting states (Li et al., 2016), and abnormal inactivation was observed in working memory tasks in patients with OSA (Prilipko et al., 2011). Previous studies have found that prefrontal CBF decreases in patients with OSA during exercise stimulation, explaining the intolerance of exercise in patients with OSA (Marillier et al., 2018). OSA is associated with loss of slow-wave sleep, and there are behavioral deficits that reflect changes in prefrontal cortex function in OSA (Nofzinger, 2005). Studies of the metabolic and cognitive consequences of sleep deprivation suggest that sleep plays a role in the restoration of prefrontal cortex function (Thomas et al., 2000). Combined with our results, the GMV atrophy of the DLPFC may be caused by poor sleep quality and sleep hypoxia, which could lead to abnormal cognitive function and psychological status, and the increased CBF in the mPFC may be a compensatory response to the GMV reduction of prefrontal lobe in OSA.

The cerebellum is considered a crucial module in motor control systems, closely related to movement and balance. It also connects to a wide range of areas of the brain, including cortex-associated and limbic regions (Schmahmann and Pandya, 1997). In this study, patients with OSA showed decreased GMV and CBF values in the cerebellum, suggesting impairment in both atrophy and blood metabolism in the cerebellum in OSA. The cerebellum is known to be susceptible to hypoxia or ischemia and is proved to be an important role in maintaining sleep, and lack of sleep may interfere with the function of the cerebellum (DelRosso and Hoque, 2014). Exposure to sleep apnea with recurrent intermittent hypoxia may be associated with abnormal GMV and CBF in the cerebellum. Several previous studies have reported decreased GMV of the cerebellum in patients with OSA when compared with HCs (Celle et al., 2009; Yaouhi et al., 2009; Joo et al., 2010; Kim et al., 2016), and Celle et al. found inverse correlations between the GMV of cerebellum and AHI scores in OSA (Celle et al., 2009), which were consistent with our results. In addition, in sensorimotor tasks, sleep deprivation interferes with functional MRI signals from many brain regions, including the cerebellum (Gazes et al., 2012). Combined with this study, the cerebellum is important for motor and cognitive networks to function properly, and reduced GMV and CBF in the cerebellum may lead to abnormal behavior in patients with OSA.

Moreover, decreased GMV in the right occipital pole, decreased CBF values in the right TP, increased CBF values in the left precentral gyrus, and the right insula were also observed in patients with OSA when compared with HCs, and the GMV loss of the occipital pole was negatively correlated with the duration of illness. Previous studies revealed reduced scattered sites of GM concentration in the temporo-parieto-occipital cortices (Yaouhi et al., 2009) and abnormal white matter volume in the occipital gyrus in patients with OSA (Huynh et al., 2014), which supported our results. After 6 weeks of continuous positive airway pressure treatment, CBF of the occipital and temporal lobe increased in patients with OSA (Maresky et al., 2019). Joo et al. (2010) reported reduced GM density in the left precentral gyrus and right insula in patients with OSA when compared with HCs, and combined with our results, we speculated that the increased CBF values of the left precentral gyrus and the right insula could be a compensatory response to GMV deficits of the left precentral and the right insula. It should be noted that GM atrophy (Joo et al., 2010; Kim et al., 2016) and hypertrophy (Fatouleh et al., 2014; Lin et al., 2016) were both found in the insula in OSA. In addition, reduced GMV of the hippocampus was reported in patients with OSA in previous studies (Morrell et al., 2003; Yaouhi et al., 2009; Canessa et al., 2011; Torelli et al., 2011; Kim et al., 2016) that did not survive in this study after FWE correction. The possible reasons accounting for these discrepancies may include small samples, demographic information, methodology (e.g., different statistical correction methods), and the duration and severity of the OSA.

The strength of this study is that it is the first time to utilize two modalities (VBM and CBF index) in patients with OSA. However, there were several limitations in this study. First, the sample size of this study was relatively small, and the findings should be verified in a larger sample study in the future. Second, as this was a cross-sectional design study, longitudinal studies such as taking CPAP therapy combined with VBM and CBF methods should be considered in the future. Finally, several potentially important new imaging markers have recently been identified that we were unable to analyze, such as cortical microinfarcts and brain atrophy.

In conclusion, this study suggested that patients with OSA had widely structural and hemometabolic abnormalities, particularly in the frontal lobe and cerebellum, and were closely related to abnormal behavior, psychology, and cognitive function, which may contribute to the pathogenesis of OSA. Large-scale, longitudinally designed randomized controlled studies are needed to further evaluate the correlation between efficacy of treatment and brain structural and blood metabolic in patients with OSA.

## Data Availability Statement

The raw data supporting the conclusions of this article will be made available by the authors, without undue reservation.

## Ethics Statement

The studies involving human participants were reviewed and approved by the Ethics Committee of the Second People’s Hospital of Guangdong Province. The patients/participants provided their written informed consent to participate in this study.

## Author Contributions

GJ designed this study and revised the manuscript. PX, KH, FC, YY, JW, XF, JY, QL, and QC contributed to data acquisition. PX contributed to data analysis and wrote the manuscript. All authors contributed and approved the final manuscript.

## Conflict of Interest

QC was employed by Philips Healthcare. The remaining authors declare that the research was conducted in the absence of any commercial or financial relationships that could be construed as a potential conflict of interest.

## Publisher’s Note

All claims expressed in this article are solely those of the authors and do not necessarily represent those of their affiliated organizations, or those of the publisher, the editors and the reviewers. Any product that may be evaluated in this article, or claim that may be made by its manufacturer, is not guaranteed or endorsed by the publisher.

## Footnotes

1
http://www.fil.ion.ucl.Ac.uk/spm

2
http://dbm.neuro.uni-jena.de/cat/

3
http://www.fil.ion.ucl.ac.uk/spm/software/spm12/
